# Qualitative modeling identifies IL-11 as a novel regulator in maintaining self-renewal in human pluripotent stem cells

**DOI:** 10.3389/fphys.2013.00303

**Published:** 2013-10-28

**Authors:** Hedi Peterson, Raed Abu Dawud, Abhishek Garg, Ying Wang, Jaak Vilo, Ioannis Xenarios, James Adjaye

**Affiliations:** ^1^Quretec Ltd.Tartu, Estonia; ^2^Faculty of Mathematics and Computer Science, Institute of Computer Science, University of TartuTartu, Estonia; ^3^Faculty of Science and Technology, Institute of Molecular and Cellular Biology, University of TartuTartu, Estonia; ^4^Molecular Embryology and Aging Group, Department for Vertebrate Genomics, Max-Planck-Institute for Molecular GeneticsBerlin, Germany; ^5^Quartier Sorge - Batiment Genopode, Swiss Institute of BioinformaticsLausanne, Switzerland; ^6^Faculty of Medicine, Institute for Stem Cell Research and Regenerative Medicine, Heinrich-Heine University DüsseldorfDüsseldorf, Germany

**Keywords:** embryonic stem cells, boolean modeling, regulatory networks, pluripotency, self-renewal

## Abstract

Pluripotency in human embryonic stem cells (hESCs) and induced pluripotent stem cells (iPSCs) is regulated by three transcription factors—OCT3/4, SOX2, and NANOG. To fully exploit the therapeutic potential of these cells it is essential to have a good mechanistic understanding of the maintenance of self-renewal and pluripotency. In this study, we demonstrate a powerful systems biology approach in which we first expand literature-based network encompassing the core regulators of pluripotency by assessing the behavior of genes targeted by perturbation experiments. We focused our attention on highly regulated genes encoding cell surface and secreted proteins as these can be more easily manipulated by the use of inhibitors or recombinant proteins. Qualitative modeling based on combining boolean networks and *in silico* perturbation experiments were employed to identify novel pluripotency-regulating genes. We validated Interleukin-11 (IL-11) and demonstrate that this cytokine is a novel pluripotency-associated factor capable of supporting self-renewal in the absence of exogenously added bFGF in culture. To date, the various protocols for hESCs maintenance require supplementation with bFGF to activate the Activin/Nodal branch of the TGFβ signaling pathway. Additional evidence supporting our findings is that IL-11 belongs to the same protein family as LIF, which is known to be necessary for maintaining pluripotency in mouse but not in human ESCs. These cytokines operate through the same gp130 receptor which interacts with Janus kinases. Our finding might explain why mESCs are in a more naïve cell state compared to hESCs and how to convert primed hESCs back to the naïve state. Taken together, our integrative modeling approach has identified novel genes as putative candidates to be incorporated into the expansion of the current gene regulatory network responsible for inducing and maintaining pluripotency.

## 1. Introduction

With the ever-growing number of experimental datasets available, the question of how to reuse data smartly to gain knowledge and advance fundamental understanding of the regulatory nature of biological systems computational methods become ever more essential. Computational methods provide a way to unravel hidden relationships and regulations between genes that would otherwise be tedious and hard to achieve with traditional approaches. Modeling and simulations should be considered as a complementary approach to the in-depth gene function analysis. Several research fields are advancing fast thanks to the improvement in technologies. Human embryonic stem cells (hESC) and induced pluripotent stem cells (iPSC) are creating major hope and research activities. These cell types are thought to be crucial to pave the way for regenerative therapies, because they are pluripotent as defined by the ability to self-renew while retaining the capacity to differentiate into all lineages of the embryo proper. This ensures both, a constant supply of cells *in vitro* and, at least equally important, the generation of donor cells for therapy. A prerequisite, however, is to understand the pluripotent state and how the undifferentiated state is maintained, as a single undifferentiated pluripotent stem cell that escapes the differentiation induction could give rise to for example tumor due to its intrinsic self-renewing characteristics. For future cell replacement therapies, it is of prime importance to be able to generate iPSCs efficiently and robustly with well defined culture conditions.

The gene regulatory network supportive of self-renewal is orchestrated by three transcription factors, namely OCT3/4, SOX2, and NANOG (Boyer et al., 2005). Pluripotency is induced in somatic cells by the over-expression of distinct combinations of these transcription factors—OCT3/4, SOX2, KLF4, and c-MYC (Takahashi and Yamanaka, 2006) or OCT3/4, SOX2, NANOG, and LIN28 (Yu et al., 2007). Although other genes are known to be associated with the main regulators as well, the overall regulatory network representing the pluripotent embryonic stem cell is still lacking completeness and potentially other regulatory mechanisms that would be able to describe both stem-cell like properties and the differentiation paths that the cells could undergo.

As mentioned above, only a small number of highly reliable transcription factors and signaling pathways active in hESCs are known (Armstrong et al., 2006; Vallier et al., 2009; Brown et al., 2011; Singh et al., 2012). Therefore, there is a need to expand and identify novel regulators, including secreted components that could be added to the culture media to sustain self renewal. Using a large set of experimental data (in our case mRNA expression data) together with *in silico* modeling methods we attempted to identify factors responsible for self renewal in human embryonic stem cells.

Previously, regulatory networks of hESCs were mostly constructed using transcription factor binding experiments or protein interaction data (Boyer et al., 2005; Wang et al., 2006; Kim et al., 2008; Muller et al., 2008). Recent developments of collecting human and mouse embryonic stem cell related experimental data sets into specialized databases like ESCDb (Jung et al., 2010) and ESCAPE (Xu et al., 2013) facilitate data reuse and combining in systematic studies of ESC regulation. Lately, Flöttmann and colleagues have shown the power of combining various experimental data sources with network modeling to gain new insights into cellular reprogramming (Flottmann et al., 2012). Boolean modeling has also been recently used by Bonzanni and colleagues to propose and validate a new regulatory connection of Gata1 regulating Fli1 in hematopoietic stem cells triggering erythrocyte lineage differentiaton (Bonzanni et al., 2013). For kinetic data and smaller network sizes stochastic modeling might be used as Kalmar and others did show how NANOG expression fluctuations mediate cell fate decisions in embryonic stem cells (Kalmar et al., 2009).

Regulatory networks are likely to be rich in different types of feedback and feedforward loops that carry the complexity of the network and provide the needed robustness of regulation (Milo et al., 2002; Remy et al., 2006; Macarthur et al., 2009). Gene regulatory networks are complex and act in more activation layers than only transcription factor and target gene level. Embryonic stem cell specific regulation can also be controlled by alternative splicing mediated regulatory switch. Gabut and others showed that FOXP1 transcription factor DNA binding domain is altered by alternative splicing of exon 18b in ESCs and that isoform targets core pluripotency regulators in hESCs (Gabut et al., 2011). Recently, Wang and colleagues identified a feedback loop active in at least two regulatory levels in hESCs. They showed that large intergenic non-coding RNA (linc-RoR) together with miR-145 and core hESC pluripotency transcription factors forms a double negative feedback loop active in ESC self-renewal (Wang et al., 2013). The identification of regulatory feedback loops are experimentally challenging and it is important to come up with strategies that can identify candidate genes and regulatory edges to expand any given cellular decision network.

In this study we use an *in silico* approach to identify novel potential regulators of undifferentiated human embryonic stem cell regulatory networks by using gene perturbation experiments together with qualitative modeling (Garg et al., 2009). Qualitative modeling suits best for large networks where edges represent both direct and indirect regulation, such as extracted from perturbation datasets, and kinetic data for all network components is difficult and even impossible to obtain. As a first step, we present a methodology for predicting new candidate genes from a given regulatory network structure. In order to identify candidate genes we set a four step approach: (i) we create a first regulatory network of the 10 core genes (e.g., *OCT3/4, SOX2, NANOG*); (ii) we identify the set of genes that were perturbed by several transcription factors and are either surface markers or secreted proteins (such as cytokines) (we retained 109 genes regulated by at least 5 out of 7 regulators); (iii) we connected these set of genes to core regulatory network (exploring all possible connections of inhibition and activation); (iv) we simulated *in silico* knockout and over-expressed each of the node and assessed through a scoring function whether the predicted network maintained pluripotency or induced differentiation. Our computational perturbation experiments revealed a new set of putative pluripotency regulating genes. We further show that one of these candidate genes, *IL-11*, supports the undifferentiated state of hESCs. Moreover, IL-11 is capable of substituting bFGF in the culture media. To date bFGF is considered irreplaceable in the culture media for maintaining hESCs in pluripotent state.

The activity of pathways in terms of signal transduction that maintain the undifferentiated state upstream of OCT3/4, SOX2, and NANOG or independent of this triad is at least partly mirrored in the composition of the culture media. Traditionally, hESCs are co-cultured with inactivated mouse embryonic fibroblasts (MEFs) supplemented with bFGF (FGF2) at a concentration of 4 ng/ml (Thomson et al., 1998), until Xu and colleagues established hESC culture on matrigel in the presence of MEF-conditioned medium (CM). However, it also needs the supplementation with bFGF (Xu et al., 2001). Later Bigdeli and others managed to culture hESCs in matrix and feeder-free culture conditions using neonatal chondrocyte-conditioned medium (Bigdeli et al., 2008). Others reported that bFGF sustains undifferentiated proliferation of hESC by simultaneous suppression of BMP4 whereby the culture conditions can be supported by adding NOGGIN as a BMP inhibitor (Xu et al., 2005b). Later, this group developed a feeder-free and matrigel-free TeSR1 medium, containing DMEM/F12 as base medium, human serum albumin, bFGF, TGFβ1, LiCl, GABA, pipecolic acid on a combination of collagen IV, fibronectin, laminin and vitronectin including high amounts of bFGF (100 ng/ml instead of 4 ng/ml) (Ludwig et al., 2006).

In 2010 it was reported that a chimeric version of IL-6 fused to its receptor when added to hESCs and allowed their cultivation in suspension. However, this was only possible with the simultaneous application of bFGF (Amit et al., 2010). Recently, the group of James Thomson re-formulated the TeSR1 medium composed of a simpler formulation (Chen et al., 2011). This medium, which they have named E8, is based on 8 elements, instead of 18 as in TeSR1. They started component pairwise analysis and found that the withdrawal of BSA is no longer toxic when removed together with β-Mercaptoethanol. It turned out that upon removal of BSA and β-Mercaptoethanol, the ingredients pipecolic acid, GABA, LiCl, chemically defined lipids, trace elements B and C, glutathione, additional thiamine and L-glutamine no longer had positive effects. The remaining 8 factors in the E8 medium are FGF2, TGFβ, NODAL, Insulin, Selenium, Transferrin, L-Ascorbic Acid, and DMEM-F12 (Chen et al., 2011). Like this report, many research articles have been published varying slightly or introducing major variations to culture conditions. However, most protocols center around the presence of bFGF in the chemically defined media or in conditioned medium (Rosler et al., 2004; Li et al., 2005; Wang et al., 2005, 2007; Xu et al., 2005a,b; Levenstein et al., 2006; Ludwig et al., 2006; Greber et al., 2007a). bFGFs benefitial effect is due to its signaling through MEK/ERK pathway (Kang et al., 2005).

The mechanism employed by bFGF acts besides on the hESCs themselves also on the mouse embryonic feeder cells or when cultured on matrigel on hESC-derived fibroblast like support cells (autologous feeders). The feeder cells produce and release supportive factors for the maintenance of undifferentiated hESCs (Bendall et al., 2007; Greber et al., 2007a). Some of these factors are TGFB1, INHBA, IGFII activating MEK/ERK, and PI3K/AKT signaling and the BMP4 antagonists CER1 and GREM1 which collectively inhibit induction of differentiation via SMAD1/5/8. Further downstream targets of bFGF are transcription factors involved in proliferation such as c-FOS, MYC, and ETS2 (Greber et al., 2007a). Like the bFGF pathway, the inhibition of TGFβ which acts via SMAD2/3 signaling induces differentiation of both hESCs and iPSCs (Greber et al., 2008).

## 2. Results

### 2.1. Pluripotency network model based on gene perturbation experiments

The literature based regulatory network of hESCs is largely built on knowledge from (Xu et al., 2002; Matin et al., 2004; Boyer et al., 2005; James et al., 2005; Vallier et al., 2005; Babaie et al., 2007; Greber et al., 2007a; Kim et al., 2008; Xu et al., 2008; Chavez et al., 2009; Lister et al., 2009; Jung et al., 2010) on the regulation and interplay between several transcription factors (Figure 2A). First, we expanded initial literature-based regulatory network of hESC using a set of seven perturbation experiments including—NANOG, SOX2, and OCT3/4 RNAi knockdowns in human embryonal carcinoma cells (NCCIT) (Greber et al., 2007b); bFGF, BMP4 and ACTA protein stimulation in H1 embryonic stem cells (Greber et al., 2008); GADD45G over-expression in NCCIT cells (Jung et al., 2010) from our previously published Embryonic Stem Cell Database (ESCDb, Figure 2B) (Jung et al., 2010). Human embryonal carcinoma cells (hECCs) are the malignant counterpart of hESCs and are frequently used as a model system for hESCs, in particular in terms of RNAi experiments. Although more limited, most likely due to their malignant character, they do have the capacity to differentiation into several cell types, and like hESCs, they undergo symmetric self-renewal (Andrews et al., 1987; Matin et al., 2004; Bahrami et al., 2005; Josephson et al., 2007). In fact, their capacity to self-renew is governed by almost the same gene circuitries for example, OCT3/4, SOX2, NANOG, DNMT3B, DPPA2, DPPA3, DPPA4, and GABRB3 (Enver et al., 2005; Greber et al., 2007b). Given that hESCs are genetically far more difficult to manipulate and even if successful, the efficiency is dissapointingly low, hECCs are frequently employed for this purpose and in comparison with genetic manipulations in hESCs they do provide useful and complementary results.

**Figure 1:**
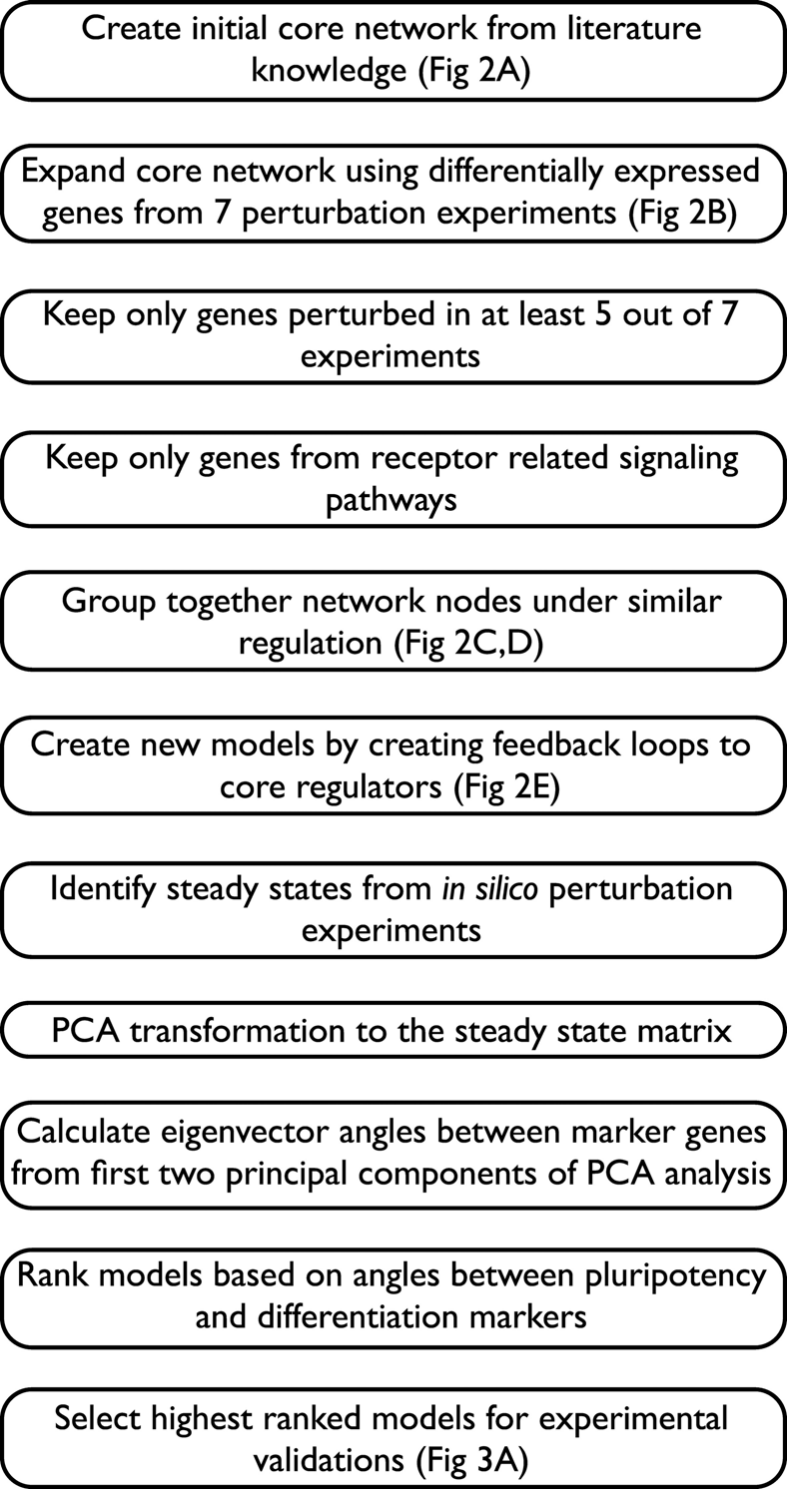
**Modeling pipeline described by individual steps for an overview of the full workflow**.

**Figure 2:**
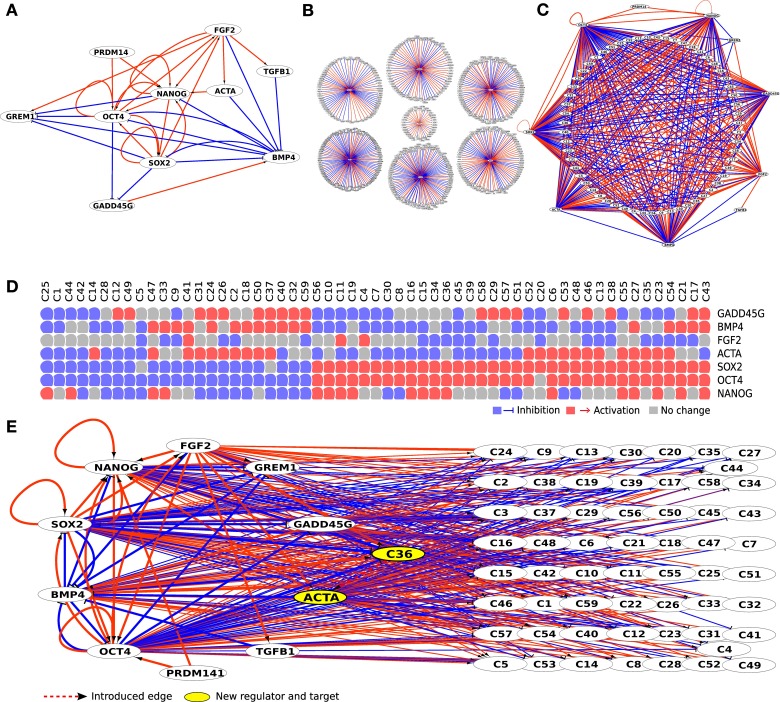
**Initial literature-based network (A) was expanded using 7 perturbation experiments from ESCDb (B)**. This resulted in a big hairball like network that was reduced by keeping only genes belonging to receptor binding GO categories (GO:0007166, GO:0005102) and having at least 5 incoming edges. All genes that had identical set of incoming edges were clustered together **(C)** with all their edges described in the panel below **(D)**. Finally, new regulatory edges were introduced creating feedback loops from clusters to regulators (dashed red line edge from C36 to ACTA) thus producing individual regulatory models **(E)**.

In the above mentioned seven perturbation experiments we identified differentially expressed genes (above and below threshold of 1.5 fold change on log2 scale) and filtered the genes based on their response to over-expression and knockdown of the selected genes (e.g., NANOG) in embryonal carcinoma and embryonic stem cells. These 7862 genes whose responses were significantly modified (either up or down-regulated) at a *p*-value of 1.0e-05 or smaller were then connected to the regulatory network in either a positive or a negative regulation (see full workflow in Figure 1). Since the expression of thousands of genes is affected by the many regulators' perturbation experiments, we concentrated on 488 genes that are affected by at least 5 perturbations, assuming that these genes are under coordinated or potentially synergistic control. We filtered genes by their Gene Ontology functional annotations and selected genes related to receptor related signaling pathways mainly because they are easier to interfere with at the protein level using either inhibitors or recombinant proteins. The 109 genes in this resulting network can be grouped together by their incoming edges, thus forming 59 clusters of genes that are under similar regulation and therefore can be grouped as one node in the final network, enabling faster computation. By grouping genes by their incoming edges we investigate the potential role of a novel feedback loop, connecting genes under a specific regulation to a regulator, in maintaining the pluripotency. Individual genes in a cluster might behave differently if studied in more detail. However, the majority of clusters contain only one or two genes and the effect of the individual genes will be evaluated separately for each gene in the experimental step. Using these steps we obtained a network composed of 69 nodes where 10 are from the core network and the rest are clusters of genes that are regulated by at least 5 out of the 7 main regulators (Figure 2C).

In the evaluation procedure we assessed the role of a regulatory edge from a terminal node (cluster of genes) to any of the core regulators (part of the core network), thus creating a new feedback loop to the network (Figure 2E). We produced two models—one with a positive and one with a negative regulation for each of the clustered genes (named C*X*, where *X* represents the cluster number, e.g., C13). In total we created a set of 1180 models where each of the introduced edges connected the 59 terminal nodes to 10 core regulators as potential targets in both positive and negative manner (59^*^10^*^2). Each model was then submitted to the boolSim (Garg et al., 2009) logical modeling simulations. boolSim produced a set of steady states for each model. We subsequently performed *in silico* knock-out and over-expression to each of the regulatory nodes contained within the models and thus obtained an ensemble of steady states that represent the effect of introduced feedback loop to pluripotency maintenance.

**Figure 3:**
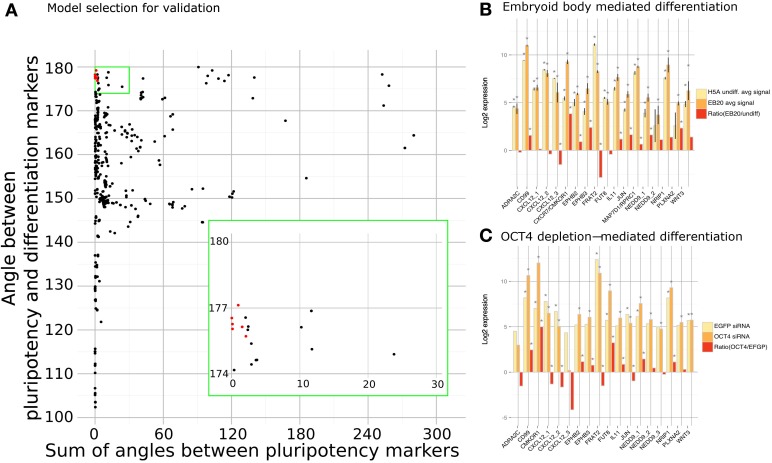
**Model scoring. (A)** Each model was scored using sum of angles between pluripotency markers (*x*-axis) and an angle between pluripotency and differentiation markers (*y*-axis). Models selected for validation (marked with red) had small angles between pluripotency markers and large angle between pluripotency and differentiation markers. Expression of each predicted factor was measured in embryoid body **(B)** and OCT3/4 **(C)** mediated differentiation. Asterisk marks significant values with *p*-values < 0.05.

We applied a PCA transformation to the matrix of steady states and identified the contributions of two classes of genes that we consider either maintaining pluripotency or inducing differentiation. Namely, we used *OCT3/4, SOX2* and *NANOG* as pluripotency marker genes and *GADD45G* and *BMP4* as differentiation markers and used their respective eigenvectors. For each model we calculated angles between the five eigenvectors in the following manner—we calculated an average angle from the three angles between SOX2 and NANOG, SOX2, and OCT3/4, and NANOG and OCT3/4; secondly, we calculated an angle between BMP4 and GADD45G; finally, we calculated the angle between the first group (NANOG, SOX2, OCT3/4) representing pluripotency and second group (BMP4, GADD45G) representing differentiation process. Models that produced small angles between pluripotency markers and at the same time a large angle between pluripotency and differentiation markers were selected as depicted in Figure 3A (selected markers marked in red). We selected the set of models that fulfills criteria of maintaining pluripotency and blocking differentiation. Using this approach we identified and selected the top 8 clusters corresponding to 15 cytokines, cytokine receptors or cytokine pathway associated factors which previously were not associated with the undifferentiated state and now appeared as candidates that might support pluripotency. We set our goal at 8 clusters mainly for feasibility reasons, but it is likely that other models might hold interesting genes that are amenable for further validation.

### 2.2. Modeling pipeline identified 15 novel cytokine pathway associated factors

These 15 novel cytokines, cytokine receptors or cytokine pathway associated factors that were predicted to support the undifferentiated state by qualitative modeling are ADRA2C, EPHB2, EPHB3, CD99, CXCL12, CXCR7, FRAT2, FUT8, IL-11, JUN, MAP7D1, NEDD9, NRIP1, PLXNA2, WNT3 (Table 1). Most of these factors have so far not been associated with the pluripotent state before. As a first step in validating our results, we checked the expression patterns of the predicted factors in studies previously reported by us (Greber et al., 2007b; Fathi et al., 2009). Indeed, we could confirm the predicted pluripotency associated factors to be differentially expressed upon embryoid body (EB)-based differentiation and, in a second data set, upon OCT3/4 depletion in hESC line H1 (Figures 3B,C). In these datasets the predicted factors can be divided into two categories. The first set is composed of genes that are down-regulated upon differentiation *CD99, CXCR7, EPHB2, EPHB3, FUT8, IL-11, MAP7D1, NEDD9, NRIP1, PLXNA2, WNT3* prompting the question whether their expression is causative, consequential, or correlative for the pluripotent state. The second set is composed of genes that are up-regulated upon differentiation, *ADRA2C, CXCL12, FRAT2*. If these genes play a role in the undifferentiated state then their up-regulated expression upon differentiation might indicate that their role in the differentiated lineages requires higher doses. JUN, however, showed contradictory behavior as it was up-regulated in the EB-based differentiation dataset and down-regulated in the OCT3/4 knockdown dataset (Figures 3B,C).

**Table 1:** **Candidate models selected for experimental validation**.

**Cluster ID**	**Candidate gene**	**Proposed regulation and target**	**Positive regulators**	**Negative regulators**
C5	WNT3	→ ACTA	ACTA, BMP4, FGF2, NANOG, OCT3/4	*none*
C14	CXCL12	→ ACTA	BMP4, GADD45G, OCT3/4, SOX2	ACTA
C15	FUT8	→ ACTA	ACTA, BMP4, FGF2	NANOG, OCT3/4, SOX2
C16	CD99	→ ACTA	ACTA, BMP4	NANOG, OCT3/4, SOX2
C16	CXCR7	→ ACTA	ACTA, BMP4	NANOG, OCT3/4, SOX2
C16	EPHB2	→ ACTA	ACTA, BMP4	NANOG, OCT3/4, SOX2
C16	EPHB3	→ ACTA	ACTA, BMP4	NANOG, OCT3/4, SOX2
C16	MAP7D1	→ ACTA	ACTA, BMP4	NANOG, OCT3/4, SOX2
C16	NEDD9	→ ACTA	ACTA, BMP4	NANOG, OCT3/4, SOX2
C16	PLXNA2	→ ACTA	ACTA, BMP4	NANOG, OCT3/4, SOX2
C28	ADRA2C	→ ACTA	ACTA, BMP4, NANOG, OCT3/4, SOX2	*none*
C36	IL11	→ ACTA	BMP4, FGF2	NANOG, OCT3/4, SOX2
C36	JUN	→ ACTA	BMP4, FGF2	NANOG, OCT3/4, SOX2
C39	NRIP1	→ ACTA	ACTA, BMP4, FGF2	OCT3/4, SOX2
C41	FRAT2	—| FGF2	OCT3/4, SOX2	ACTA, BMP4, FGF2

*Each model, consisting of cluster ID, proposed regulation and target triplet, is described with its regulators and genes belonging to the cluster*.

We then decided to test a subset of these factors and applied the recombinant form of these cytokines or inhibitors of the respective pathways to human ESCs. First, we tested various concentrations of recombinant cytokines EPHB2 and EPHB3 (100 ng/ml–5 μg/ml each)(data not shown), WNT3A (1–100 ng/ml), IL-11 (1–100 ng/ml), on undifferentiated hES cell line H1 in N2B27 medium (Supplementary Figure 1). We also added the inhibitors Chalcone4 (10 nM–10 μM) that inhibits CXCL12 and thereby disables the CXCL12-CXCR7 axis. In order to inhibit JUN we added SP600125 (0.1–50 μM) which does inhibit JUN indirectly by the inhibition of its kinase and activator, C-JUN N-terminal-Kinase (JNK). After 3 days the cells were stained for Alkaline Phosphatase (AP) (Supplementary Figure 1). Three days of bFGF deprivation was sufficient to initiate differentiation. No effects were observed upon stimulation with WNT3A, EPHB2, EPHB3, and Chalcone4. However, the SP600125 inhibitor showed a cytotoxic effect at 30 and 40 μM and dramatic toxic effects at 50 μM (Supplementary Figure 1). At 10 and 20 μM some toxic effects were only observed in N2B27 medium, but not in MEF-conditioned medium. The addition of IL-11 induced the strongest AP staining at 10 ng/ml.

### 2.3. IL-11 is a novel pluripotency supporting gene

**Figure 4:**
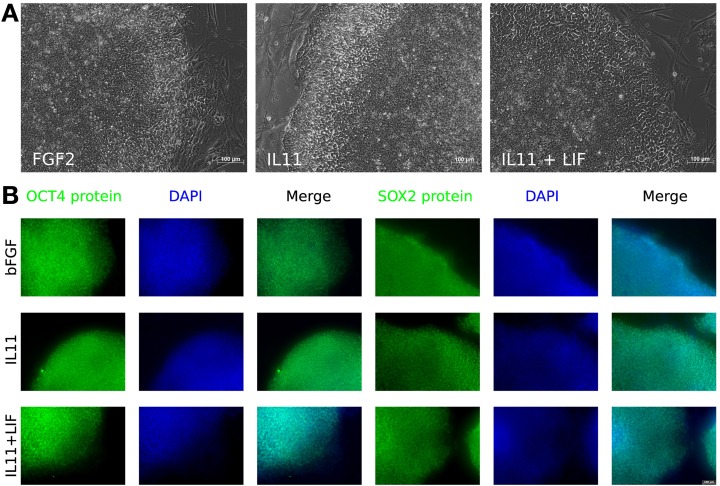
**IL-11 treatment. (A)** Morphology of hESCs maintained with different cytokines—FGF2 (left), IL-11 (middle), and IL-11 plus LIF (right). **(B)** Immunocytochemistry for OCT3/4 (columns 1–3) and SOX2 (columns 4–6) on bFGF treated cells (top row), IL11 treated cells (middle row) and IL11 plus LIF treated cells (bottom row).

**Figure 5:**
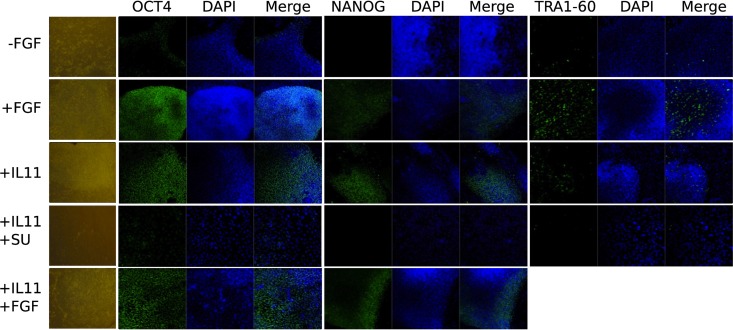
**Immunocytochemistry and phase contrast showing the maintenance of pluripotency marker expression OCT3/4, NANOG, and TRA1-60 upon IL11 application compared to FGF2 positive and negative control and autocrine effect of FGF2 by blocking the FGF receptor with SU5402 after 3 days**.

We focused further investigations on IL-11, because treatment of hESCs with this cytokine exhibited the most promising effect. The cells were stepwise adapted to grow in medium supplemented only with IL-11 and cultured on MEFs as feeder cells. First, the cells were cultured in 50% MEF-conditioned medium (CM) and 50% unconditioned hES medium (UM). This medium mixture was supplemented with 10 ng/ml IL-11 instead of bFGF and cultured for two passages. Then, cultured for another two passages with 10%:90% (CM:UM) and finally in UM only supplemented with 10 ng/ml IL-11. This strategy succeeded in our hands only when combined with the application of the ROCK inhibitor and Thiazovivin, simultaneously. The cells were grown in IL-11 hESC medium for at least 9 passages not only maintaining the undifferentiated morphology, but also immunohistochemistry experiments showed that the cells were positive for OCT3/4, SOX2, NANOG, and TRA1-60 (Figures 4, 5).

In order to understand the signaling mechanism of IL-11 in comparison to bFGF signaling in supporting the undifferentiated state we employed microarray-based gene expression profiling. The hESCs line H9 culture was deprived of bFGF for 5 days and as negative control compared with the bFGF supplemented positive control and IL-11 supplementation as the test of our prediction. Although 5 days were not long enough to induce dramatic morphological change upon bFGF withdrawal (Figure 4) early effects of differentiation could already be studied. OCT3/4 and NANOG immunohistochemistry showed intermediate signal intensities in the IL-11 treated cells in comparison to the negative and positive controls (Figure 5) (full data available as Supplementary Table 1). The global gene expression as illustrated in the correlation data (Supplementary Table 2) and hierarchical clustering mirrored these results. The largest differences appeared between the bFGF supplemented positive control and the bFGF deprived negative control, *R*^2^ < 0.948 (Supplementary Table 2). The gene expression profile of the IL-11 treated cells occupied an intermediate state that is slightly closer to the bFGF deprived control, 0.9838 > R^2^ > 0.9737 (correlation to bFGF supplemented control 0.9509 > R^2^ > 0.9411). Similarly, the hierarchical clustering reveals also that the IL-11 treatment occupies an intermediate state. The fact that the *R*^2^ value of the IL-11 treatment is closer to that of bFGF deprived control than the bFGF treatment might hint toward a potentially distinct mechanism being employed by IL-11 than by bFGF in supporting the pluripotent state.

**Figure 6:**
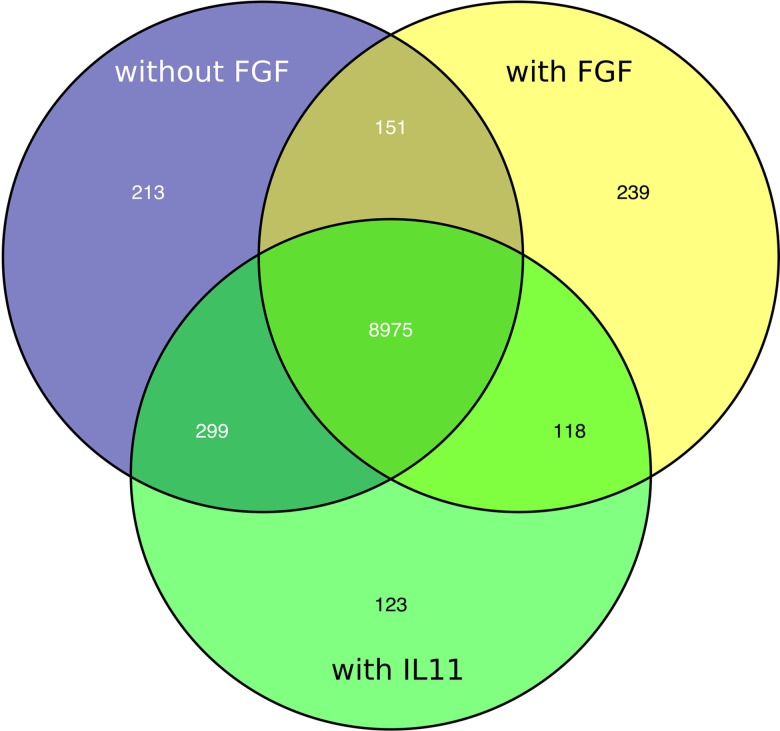
**Venn diagram showing distinct and overlapping transcriptomic signatures of FGF2 (yellow), IL11 (green) treated cells and non-treated cells (blue)**.

The Venn diagram shows the overlapping and distinct transcriptome signatures whereby 8975 genes were expressed in all three samples −bFGF, +bFGF and +IL-11 (Figure 6). Amongst the common expressed genes were also pluripotency markers like OCT3/4, SOX2, NANOG, because 5 days are not long enough to induce full differentiation, but long enough to identify putative direct targets of IL-11. However, in the +bFGF sample OCT3/4 was more than 2.6 fold higher expressed and NANOG more than 1.7 fold higher expressed than in the −bFGF sample indicating that the differentiation process had been inititated. 239 genes were distinctly expressed in the bFGF treatment and 123 genes upon IL-11 treatment. Another 118 genes were commonly expressed in the bFGF and in the IL-11 treatment. The lists of the distinct Venn diagram compartments are found in Supplementary Table 3. KEGG pathway analysis revealed that the common genes expressed in FGF2 and IL-11 are enriched for cell adhesion, tight junction, insulin signaling and cancer pathways. In the IL-11 only compartment we found enrichment for regulation of actin cytoskeleton and adherens junction (Tables 2, 3). Since IL-11 belongs, like LIF, to the IL-6 family and both signal via gp130 as co-receptor we tested whether the addition of murine LIF would have a beneficial effect. We did not observe morphological differences between IL-11+LIF and IL-11 only treated cells nor differences in intensities of immunostainings for OCT3/4 and SOX2 (Figures 4A,B).

**Table 2:** **KEGG Pathway involvement in commonly expressed genes in FGF2 and IL-11 treatments**.

**Pathway**	**Gene count**	***p*-value**	**Genes**
Cell adhesion moleculs (CAM)	4	0.05703	SDC1, CD34, CLDN10, ICOSLG
Tight junction	4	0.05915	INADL, CLDN10, AKT3, CTNNB1
Insulin signaling pathway	4	0.06022	TSC1, PRKAR1A, SOCS4, AKT3
Pathways in cancer	6	0.06459	PDGFA, PML, FGF12, COL4A6, AKT3, CTNNB1
Melanoma	3	0.08100	PDGFA, FGF12, AKT3

**Table 3:** **KEGG pathway involvement enriched for genes expressed in IL-11 treatments only**.

**Pathway**	**Gene count**	***p*-value**	**Genes**
Regulation of actin cytoskeleton	5	0.05345	PFN2, WASF1, MRAS, ITGA1, ACTN2
Adherens junction	3	0.09307	WASF1, ACTN2, FARP2

## 3. Discussion

In this study we used a combination of literature knowledge and experimental datasets to expand regulatory network of human embryonic stem cells. Since the identification of core regulatory triplet of hESCs by (Boyer et al., 2005) many groups have composed pluripotency networks (Muller et al., 2008; Chavez et al., 2009; Lister et al., 2009; Macarthur et al., 2009; Won et al., 2012). Most of these networks have been composed using co-expression, protein-protein interaction or chromatin-immunoprecipitation studies and more for descriptive than modeling purposes. In this paper, we used perturbation experiments that reflect regulatory protein impact on its targets. By doing that we could evaluate the potential of using only perturbation datasets for reconstructing a gene regulatory network as other data types such as protein-protein interactions or chromatin immunoprecipitation data might be harder to obtain. While connections between genes in this type of a network cannot be distinguished between direct and indirect, we can expect the differentially expressed genes to be downstream targets of the perturbed genes. The nature of these datasets suit perfectly for boolean modeling as we just need to define potential direct or indirect regulation between gene-gene pairs and do not need kinetic constants that are hard to acquire for a larger gene set. Although perturbation experiments have been previously used by (Amit et al., 2009) to study how human pathogen-sensing pathways acquire specifity, it is to our knowledge the first time this approach has been used for expanding pluripotency governing networks in pluripotent cells. Of course perturbation-based network can be combined besides literature knowledge also with other types of gene-gene interaction expressing datatypes and with the ever-expanding data sources it would be potential next step to study the complexity of pluripotency.

Perturbation experiments are helpful in identifying downstream targets of the affected gene and therefore a promising approach for identifying gene targets in a high-throughput manner. However, it is not possible to do all the potential perturbations for a given cellular system *in vitro* as the proteins might be difficult to over-express or the RNAi needed are not known for genes of interest. Here, *in silico* modeling comes into play as the computational perturbation experiments are easy to execute with a network modeling approach. In this work we created network models to select a handful of candidate genes to be experimentally validated from hundreds of cytokine pathway associated factors that otherwise would have been hard to prioritize.

In order to score the created models we used angles between eigenvectors from principal component analysis of network activity states. When the angles are small between eigenvectors then the variables, in our case pluripotency markers, are correlated. We used this approach as it is known that the core regulatory circuit of OCT3/4, SOX2, and NANOG have to be all active in order to maintain pluripotency and inactivate differentiation-inducing signals.

Out of the prioritized 15 novel factors that our method predicted we were able to either supplement or inhibit only 6 genes (CXCL12, EPHB2, EPHB3, IL-11, JUN, and WNT3A) as for these genes we were able to find recombinant proteins or known inhibitors. Out of these 6 genes, we were able to show that IL-11 plays an important role in maintaining pluripotency in hESCs. A probable reason why the other 5 genes that we were able to test did not exert such a strong effect might be either that the concentrations used were sub-optimal or that their effect is not potent enough to maintain pluripotency. In case of EPHB2 and EPHB3, which are membrane bound ligands and are involved in bi-directional signaling, might not fulfill our predicted function when supplemented in a soluble form as we did.

The known critical genetic factors that govern the pluripotent state in the murine and human system are conserved to a large extent. For example, the core transcription factors OCT3/4, SOX2, and NANOG are common to both, the murine and the human system (Chambers et al., 2003; Boyer et al., 2005; Ying et al., 2008; Jung et al., 2010) as well as other factors like KLF4, c-MYC are expressed in both cell types. In fact, their reactivation in generating induced pluripotent stem cells is essential in both systems (Takahashi and Yamanaka, 2006; Wernig et al., 2007). However, it seemed that there are also differences between mESCs and hESCs. For example the role of signaling pathways is less conserved. In murine embryonic stem cells (mESCs) the LIF/STAT3 pathway can sustain self-renewal but in serum free conditions the additional activity of BMP4 is required. LIF independence can be achieved by FOXP1 ESC specific splice isoform (Gabut et al., 2011) or by inhibition of the ERK branch of FGF signaling using the PD0325901 inhibitor supported by inhibition of GSK3 using CHIR99021 which helps to stabilize β-CATENIN (Ying et al., 2008). The latter, therefore, attributes supportive effects of WNT signaling to the pluripotent state. Although, mESCs and hESCs were considered different in signaling activites, similar observations were made in hESCs using BIO, another GSK inhibitor. However, this was only possible in the presence of FGF2 (Sato et al., 2004). In the hESCs, the LIF/STAT3 pathway fails to show any supportive effect, and in contrast to the murine ESCs the ERK signaling is required to mediate bFGF, PDGF, S1P signaling. Further SMAD2/3 (activated through TGFβ), PI3K/AKT signaling (activated through IGFII) and NFκβ signaling are essential to maintain the pluripotent state as judged by down-regulation of these pathways upon embryoid body formation and other functional experiments (Armstrong et al., 2006; Bendall et al., 2007; Greber et al., 2008). BMP4 induces trophectoderm differentiation acting via SMAD1/5/8 (Xu et al., 2005b; Sudheer et al., 2012).

These signaling differences became somewhat clearer when it was realized that mESCs represent a more “naïve” state than the “primed” mESCs and hESCs (Brons et al., 2007; Tesar et al., 2007). It is possible to isolate pluripotent murine stem cells from the ICM and the post-implantation epiblast, a developmentally later stage. These so called and primed EpiStemCells (EpiSC) behave very similar to hESCs, ranging from flat colony morphology, non-responsive to LIF, and dependence on FGF2 and ACTIVIN A, sensitivity to single-cell dissociation, X-chromosome inactivation (XCI), low chimera-contribution (Tesar et al., 2007; Ying et al., 2008; Hanna et al., 2010; Lengner et al., 2010), etc. Hence, it appears that the differences between the mESCs and hESCs are not due to species differences, but are attributable to different developmental stages. Therefore, it seems justified to speculate that a human naïve pluripotent counterpart exists. Indeed, Hanna and colleagues showed for a short period of twenty passages that it was possible to force human pluripotent stem cells to adopt a naïve phenotype (Hanna et al., 2010). mEpiSCs, which are similar to hESCs, require FGF/ERK signaling for both maintaining the pluripotent state and preventing from falling back into the naïve state (Kunath et al., 2007; Nichols et al., 2009; Lanner and Rossant, 2010). Identifying IL-11 as a pluripotency promoting factor in human ESCs renders the signaling differences between the human and murine pluripotent systems that are largely accentuated by the role played by LIF in the murine naïve, but not in the human “primed” system, as less pronounced as previously thought. Nonetheless, for other mechanisms it is known that they act in synergy, e.g., on establishing pregnancy (Dimitriadis et al., 2010).

Our discovery that IL-11 can replace exogenous FGF2 may represent a stepping stone for interconverting the two pluripotent states, naïve and primed, and thereby potentially be the first step from releasing the primed cells from bFGF dependency to pave the way for de-differentiation to the naïve state. In line with this hypothesis is that LIF and IL-11 belong to the IL-6 family and signal through the same co-receptor gp130 (Nandurkar et al., 1996; Timmermann et al., 2000). Although in our study we did not observe any effects upon LIF treatment, we cannot exclude a potential role for LIF, because we applied the murine LIF (mLIF) instead of the human LIF (hLIF). Both are highly conserved, but there are slight variations which could affect their function. In fact, Daheron and colleagues showed that hLIF leads to STAT3 phosphorylation in mESCs while mLIF fails to activate human STAT3 (Daheron et al., 2004). Further, the study of Hanna and others showed at least transionary that hLIF can support the human pluripotent state (Hanna et al., 2010).

If IL-11 indeed aids in the conversion of the primed to the naïve state or stabilizes the primed pluripotent state, then it seems incapable of fulfilling this role on its own. It might require the support from other factors for several reasons. First, the IL-11 treatment resulted in a gene expression signature that is closer to the FGF untreated state and the expression intensities of OCT3/4 and SOX2 are intermediate as well. Second, morphologically we did not observe colony similarities to the naïve state. Third, the IL-11 treatment is hypersensitive to single cell dissociation, as judged by the requirement of ROCK and Thiazovivin simultaneously. Presumably, it's also still in an X_*a*_X_*i*_ state instead of an X_*a*_X_*a*_ which has to be addressed experimentally.

The question still remains, what are the mechanisms employed by IL-11 and FGF2 to sustain the pluripotent state? The mechanism employed by bFGF acts on the mouse embryonic feeder cells or when cultured on matrigel on the hESC derived fibroblast like support cells (autologous feeders) to produce and release supportive factors for the maintenance of undifferentiated hESCs (Greber et al., 2007a). Some of these factors are TGFB1, INHBA, IGFII (in order to activate MEK/ERK and PI3K/AKT signaling) and the BMP4 antagonists CER1 and GREM1 to avoid induction of differentiation via SMAD1/5/8 (Bendall et al., 2007; Greber et al., 2008). Further downstream targets of bFGF are transcription factors involved in proliferation such as c-FOS, c-JUN, MYC, and ETS2 (Greber et al., 2007a). Also IL-11 was up-regulated which is traditionally known to be involved in proliferation in the hematopoetic system (Nandurkar et al., 1998). Like the bFGF pathway, the inhibition of TGFβ which acts via SMAD2/3 signaling induces differentiation in hESCs (Greber et al., 2008), PI3K/AKT signaling is repressed upon embryoid body formation (Armstrong et al., 2006) and it was discovered that IGFII initiates this signaling pathway by binding to IGFR1 (Wang et al., 2005). In the same study, the authors demonstrated that the IGFII pathway seems to be more dominant than the bFGF pathway. They demonstrated that the addition of IGFII and FGFR inhibitor could maintain the undifferentiated state whereas vice versa it was not possible. The pathways of PI3K/AKT and MEK/ERK cross-talk through GSK3, because AKT phosphorylates and inactivates GSK3α and GSK3β. Also, the PI3K/AKT pathway cross-talks with the NFκβ pathway. AKT phosphorylates Iκβ, the cytosolic inhibitor of κβ (NFκβ). There are indications that the NFκβ pathway acts upstream of the MEK/ERK pathway. IL-11 seems to employ different mechanisms, as our data indicate. The *R*^2^ values of the IL-11 treatment are closer to the bFGF deprived control than the bFGF treatment. In terms of gene expression, this might be a hint of a global dimension that a distinct mechanism is being employed. More specific mechanisms are indicated by the activated pathways of adherens junction and the actin cytoskeleton, which was implicated by Thomson and his colleagues (Chen et al., 2010) to be associated with the pluripotent state. The cytoskeletal involvement is very clear when the ROCK inhibitor is used on single cell suspension blocking the myosin-actin mediated contraction of hESC and thus ensuring survival (Chen et al., 2010).

It appears that the bFGF and IL-11 mediated promotions of pluripotency belong to independent mechanisms and it might be justified to speculate that the combined application of both might lead to additive reinforcement of self-renewal and pluripotency (which ever this might be—naïve or primed) by stabilizing MEK/ERK and the activation of the actin cytoskeleton pathway, respectively. However, in order to convert hESCs into a naïve state or to stabilize the primed state even further in a FGF2-independent manner, additional supporting cytokine, inhibitors or conditions need to be found to strengthen the effect of IL-11, because to date it appears as if bFGF traps the primed pluripotent state by preventing differentiation and further de-differentiation (Thomson et al., 1998; Li et al., 2009; Greber et al., 2010; Hanna et al., 2010). These insights will have substantial implications both on the iPS cells and deepen our understanding of pluripotent substates. Taken together our study shows that our combined approach of systems biology and experimental biology can identify factors that are counterintuitive and, hence, hard to discover otherwise. Equally important, this approach can be applied to any other cellular system and thereby enhance research progress.

## 4. Materials and methods

### 4.1. Identification of candidate genes using qualitative modeling

The initial literature based network was very limited and composed of bFGF/MEK/ERK/NANOG (Xu et al., 2008) signaling as well as TGFβ1, ACTIVIN, NODAL/SMAD2/3/NANOG (Xu et al., 2008) in competition with BMP4/SMAD1/5/8 which drives differentiation (Xu et al., 2002). This configuration of signaling cascades was thought to maintain the expression of the key factors of the pluripotent state OCT3/4, SOX2, and NANOG which are transcription factors that form multiheteromers in different combinations, but also bind their target genes individually (Boyer et al., 2005). Knock down experiments, i.e., of OCT3/4, showed that hCG and GCM1 are directly or indirectly negatively regulated by OCT3/4 (Matin et al., 2004). In addition GADD45G and BMP4 have been shown to be negatively regulated by OCT4 (Babaie et al., 2007) and are direct targets of OCT4 (Jung et al., 2010).

We expanded the initial literature network by using gene perturbation data from Embryonic Stem Cell Database (Jung et al., 2010). *OCT3/4, SOX2*, and *NANOG* RNAi knockdowns in human embryonal carcinoma cells (NCCIT) are from (Greber et al., 2007b). Protein supplementation experiments of bFGF, BMP4, and ACTIVIN A in human H1 embryonic stem cells originates from (Greber et al., 2008) and GADD45G addition experiment in NCCIT cells was described by (Jung et al., 2010) (Figure 2B).

Edges in the expanded network were defined by the perturbation expriments in the following manner: genes that showed significant expression change (at least 1.5 fold change in log2 scale with *p*-value less than 1.0e-05; detection *p*-value less than 1.0e-05) after the perturbation were added to the network. Genes showing up-regulation after adding the protein gained positive incoming edge, while genes showing down-regulation gained negative incoming edge. The opposite applies for negative perturbation experiments of RNAi knockdowns.

In order to limit the search space of possible important regulators, we first concentrated on genes that had at least five incoming edges from the core regulators. This enabled to keep only these genes that are under strong regulation of known regulators and therefore more likely to be important for given cellular states. Secondly, we considered only genes that are either receptors or ligands by including only genes belonging to cell surface receptor linked signaling pathway (GO:0007166) and receptor binding (GO:0005102) Gene Ontology categories. This filtering served the purpose to identify candidate genes that could be more easily validated experimentally. Finally, genes having identical incoming regulatory edges were clustered together to a node in order to reduce the network size, thus forming a reduced perturbation network (Figure 2C). All possible network sizes and respective running times are shown in Supplementary Table 4.

We composed models by introducing a new regulatory edge to the reduced perturbation network. The edge is added from a terminal node (cluster, having only incoming edges) to any regulator (nodes from initial literature network having outgoing edges) in the network. The edge can be either activating or inhibiting. Each model represents a network that differs from the reduced perturbation network by only an added edge (Figure 2E).

Using these model networks we performed *in silico* perturbation experiments using boolSim (version 1.0) program developed by Garg et al. (2009). boolSim was run in synchronous mode where all nodes change values simultaneously in sequential time points. We did not set a limit for the number of steps to be done before falling into steady states. The parameters we used were *-p* with value 1 for synchronous mode; *-f* for input network file; *-o* for output of identified steady states; *-e* specifying the perturbed node and type of perturbation (over-expression or knockdown). We performed for all regulatory nodes in each model network both over-expression and knockdown experiments. Each *in silico* perturbation experiment leads to zero or more steady states which express the potential activation and inactivation status of our model network. We consider as steady states both fixed and strongly connected steady states, where subsets of the network nodes might be in cyclic steady states. Such states can be described as binary vectors where 1 marks active and 0 a passive mode of each node in the network. These vectors were next merged into a steady state matrix where each row represents one steady state and each column represents a node in the network.

In order to score the models we defined OCT3/4, SOX2, and NANOG as pluripotency marker genes. As differentiation markers we used BMP4 as it is known to induce the differentiation of human ES cells to trophoblast (Xu et al., 2005b) and GADD45G that is known to activate differentation pathways (Jung et al., 2010).

In the assessment step we reduced the dimensions of the steady state matrix with principal component analysis using the latest version of prcomp package (Venables and Ripley, 2002) in R version 2.13.0 (R Development Core Team, 2011). In this analysis the first principal component, PC1, represents the direction of the highest variance of the data and the second principal component, PC2, represents the highest remaining variance orthogonal to the first component. We used the first two principal components to define a vector for each marker gene and calculated angles between such vectors to score our model networks. Resulting angles are small between same state markers (either pluripotency markers *OCT3/4, SOX2*, and *NANOG*; or differentiation markers *BMP4* and *GADD45G*) and large between different states when the model represents a regulatory network where same cell state markers (e.g., *BMP4* and *GADD45G*) are in one state (e.g., inactivated) and the second state markers (e.g., *OCT3/4, SOX2*, and *NANOG*) are in the opposite state (e.g., activated). Each of the models is therefore scored by three different angles and models having minimal angle between pluripotency markers and having maximal angle between pluripotency markers and differentiation markers were selected for experimental testing (Figure 3A).

### 4.2. Cell culture

Human ESCs were maintained on Mytomycin C inactivated mouse MEFs in KnockOut DMEM (Invitrogen) supplemented with 20% KnockOut serum replacement (Invitrogen), 0.1 mM non-essential amino acids (Invitrogen), 1 mM L-glutamine (Invitrogen), 0.1 mM β-mercaptoethanol (Sigma), 8 ng/ml bFGF (Invitrogen). For passaging, hESCs and cells were split 1:3 using 1 mg/ml collagenase IV (Invitrogen). Alternatively, for feeder-free culture, hESCs cells were plated on Matrigel (Becton Dickinson)-coated plate in MEF-conditioned medium supplemented with 8 ng/ml bFGF or in N2B27 medium supplemented with 24 ng/ml bFGF. The SP600125 (Sigma) inhibitor was dissolved in DMSO and added to the cells to a final concentration of 5 or 10 μM. Recombinant IL-11 (Sigma) was added to a final concentration of 10 ng/ml and murine LIF 10^3^ Units/ml (Millipore).

### 4.3. Alkaline phosphatase staining and immunofluorescence staining

Alkaline phosphatase staining was performed using the alkaline phosphatase detection kit (Millipore) according to the manufacturer's protocol. For immunofluorescence staining, the cells were fixed in 4 % paraformaldehyde in PBS for 15 min, permeabilized using 0.1 % Triton X-100 (Sigma) for 30 min at RT and blocked with 5% chicken or fetal calf serum for 30 min. Subsequently, the cells were incubated with the primary antibodies in blocking solution overnight at 4°C. The primary antibodies included monoclonal antibodies against OCT3/4 (Santa Crutz, 1:100), SOX2 (Santa Cruz, 1:100), NANOG (Abcam, 1:500), SSEA-1 (Millipore, 1:500), SSEA-3 (Abcam, 1:500), SSEA-4 (Abcam, 1:500), TRA-1-60 (Millipore, 1:500), TRA-1-81 (Millipore, 1:500).

### 4.4. Microarray analysis

Total RNA was extracted using the MiniRNeasy Kit according to the manufacturer's protocol. The quality was checked by Nanodrop analysis (Nanodrop Technologies). 500 ng were used for biotin-labeled cRNA production using a linear amplification kit (Ambion). Hybridizations, washing, Cy3-streptavidin staining, and scanning were performed on the Illumina BeadStation 500 platform (Illumina) according to manufacturer's instruction. cRNA samples were hybridized onto Illumina human-8 BeadChips version 3. All basic gene expression data analyses were carried out using the BeadStudio software 3.0. Raw data were background-subtracted, normalized using the *rank invariant* algorithm and then filtered for significant expression on the basis of negative control beads. Pathway analyses were determined according to Gene Ontology terms or mapped to KEGG pathways (Kanehisa et al., 2012) and BIOCARTA pathways (http://www.biocarta.com/genes/index.asp) with DAVID 2006 (Huang et al., 2009a,b) using GenBank accession numbers represented by the corresponding chip oligonucleotides as input. Then gene expression arrays (Illumina) for genome-wide expression analysis was carried out. The expression data have been deposited in the Gene Expression Omnibus (GEO) database, www.ncbi.nlm.nih.gov/geo (accession no. GSE51159).

### 4.5. Statistical analysis

A groups comparison of two were made using Student's two-tailed *t*-test. Data represent the mean of triplicates and are expressed as mean and standard deviation. All statistical analyses were performed using the SPSS 6.0 statistical software program. *P*-values of < 0.05 were considered statistically significant.

## Author contributions

Hedi Peterson performed the modeling and wrote the manuscript; Raed Abu Dawud performed all biological experiments and wrote the manuscript; Abhishek Garg provided the modeling software and participated in designing the modeling pipeline; Ying Wang performed some biological replicate experiments; Jaak Vilo, Ioannis Xenarios, James Adjaye initiated, supervised the work and edited the manuscript.

### Conflict of interest statement

The authors declare that the research was conducted in the absence of any commercial or financial relationships that could be construed as a potential conflict of interest.

## Supplementary material

Supplementary Figure 1 **Raw data of initial alkaline phosphatase screen of predicted candidate genes for 3 days**. Shown are four 12 well plates, each testing various concentrations of the predicted candidate cytokines or inhibitors of the respective pathway. Top left, WNT3A 1–100 ng/ml. Top right, Chalcone4 (inhibiting the CXCL12—CXCR7 axis) 10 nM–10 μM. Bottom left, IL-11 1–500 ng/ml. Bottom right, SP600125 (inhibiting JNK which in turn fails to activate c-JUN) tested at concentrations ranging from 0.1 to 50 μM. Pink arrows show DMSO control, red arrows show water control and the green arrows the SP600125 at 50 μM.

Supplementary Table 1 **Illumina expression data of ES cells treated with and without FGF2 and IL-11**.

Supplementary Table 2 **The global gene expression as illustrated in the correlation data**.

Supplementary Table 3 **The lists of the distinct Venn diagram compartments**.

Supplementary Table 4 **Various network sizes and corresponding calculation times**.

## Research Topic:

From structural to molecular systems biology: experimental and computational approaches to unravel mechanisms of kinase activity regulation in cancer and neurodegeneration

## Topic Editor:

Matteo Barberis, Humboldt University Berlin, Germany; Max Planck Institute for Molecular Genetics, Berlin, Germany

## References

[B1] Amit I. Garber M. Chevrier N. Leite A. P. Donner Y. Eisenhaure T. (2009). Unbiased reconstruction of a mammalian transcriptional network mediating pathogen responses. Science 326, 257–263 10.1126/science.1179050 19729616 PMC2879337

[B2] Amit M. Chebath J. Margulets V. Laevsky I. Miropolsky Y. Shariki K. (2010). Suspension culture of undifferentiated human embryonic and induced pluripotent stem cells. Stem Cell Rev. 6, 248–259 10.1007/s12015-010-9149-y 20431964

[B3] Andrews P. W. Fenderson B. Hakomori S. (1987). Human embryonal carcinoma cells and their differentiation in culture. Int. J. Androl. 10, 95–104 10.1111/j.1365-2605.1987.tb00170.x 2438234

[B4] Armstrong L. Hughes O. Yung S. Hyslop L. Stewart R. Wappler I. (2006). The role of PI3K/AKT, MAPK/ERK and NFkappabeta signalling in the maintenance of human embryonic stem cell pluripotency and viability highlighted by transcriptional profiling and functional analysis. Hum. Mol. Genet. 15, 1894–1913 10.1093/hmg/ddl112 16644866

[B5] Babaie Y. Herwig R. Greber B. Brink T. C. Wruck W. Groth D. (2007). Analysis of Oct4-dependent transcriptional networks regulating self-renewal and pluripotency in human embryonic stem cells. Stem Cells 25, 500–510 10.1634/stemcells.2006-0426 17068183

[B6] Bahrami A. R. Matin M. M. Andrews P. W. (2005). The CDK inhibitor p27 enhances neural differentiation in pluripotent NTERA2 human EC cells, but does not permit differentiation of 2102Ep nullipotent human EC cells. Mech. Dev. 122, 1034–1042 10.1016/j.mod.2005.04.011 16023837

[B7] Bendall S. C. Stewart M. H. Menendez P. George D. Vijayaragavan K. Werbowetski-Ogilvie T. (2007). IGF and FGF cooperatively establish the regulatory stem cell niche of pluripotent human cells *in vitro*. Nature 448, 1015–1021 10.1038/nature06027 17625568

[B8] Bigdeli N. Andersson M. Strehl R. Emanuelsson K. Kilmare E. Hyllner J. (2008). Adaptation of human embryonic stem cells to feeder-free and matrix-free culture conditions directly on plastic surfaces. J. Biotechnol. 133, 146–153 10.1016/j.jbiotec.2007.08.045 17935814

[B9] Bonzanni N. Garg A. Feenstra K. A. Schutte J. Kinston S. Miranda-Saavedra D. (2013). Hard-wired heterogeneity in blood stem cells revealed using a dynamic regulatory network model. Bioinformatics 29, i80–i88 10.1093/bioinformatics/btt243 23813012 PMC3694641

[B10] Boyer L. A. Lee T. I. Cole M. F. Johnstone S. E. Levine S. S. Zucker J. P. (2005). Core transcriptional regulatory circuitry in human embryonic stem cells. Cell 122, 947–956 10.1016/j.cell.2005.08.020 16153702 PMC3006442

[B11] Brons I. G. Smithers L. E. Trotter M. W. Rugg-Gunn P. Sun B. Chuva de Sousa Lopes S. M. (2007). Derivation of pluripotent epiblast stem cells from mammalian embryos. Nature 448, 191–195 10.1038/nature05950 17597762

[B12] Brown S. Teo A. Pauklin S. Hannan N. Cho C. H. Lim B. (2011). Activin/Nodal signaling controls divergent transcriptional networks in human embryonic stem cells and in endoderm progenitors. Stem Cells 29, 1176–1185 10.1002/stem.666 21630377

[B13] Chambers I. Colby D. Robertson M. Nichols J. Lee S. Tweedie S. (2003). Functional expression cloning of Nanog, a pluripotency sustaining factor in embryonic stem cells. Cell 113, 643–655 10.1016/S0092-8674(03)00392-1 12787505

[B14] Chavez L. Bais A. S. Vingron M. Lehrach H. Adjaye J. Herwig R. (2009). *In silico* identification of a core regulatory network of OCT4 in human embryonic stem cells using an integrated approach. BMC Genomics 10:314 10.1186/1471-2164-10-314 19604364 PMC2714862

[B15] Chen G. Gulbranson D. R. Hou Z. Bolin J. M. Ruotti V. Probasco M. D. (2011). Chemically defined conditions for human iPSC derivation and culture. Nat. Methods 8, 424–429 10.1038/nmeth.1593 21478862 PMC3084903

[B16] Chen G. Hou Z. Gulbranson D. R. Thomson J. A. (2010). Actin-myosin contractility is responsible for the reduced viability of dissociated human embryonic stem cells. Cell Stem Cell 7, 240–248 10.1016/j.stem.2010.06.017 20682449 PMC2916864

[B17] Daheron L. Opitz S. L. Zaehres H. Lensch M. W. Lensch W. M. Andrews P. W. (2004). LIF/STAT3 signaling fails to maintain self-renewal of human embryonic stem cells. Stem Cells 22, 770–778 10.1634/stemcells.22-5-770 15342941

[B18] Dimitriadis E. Menkhorst E. Salamonsen L. A. Paiva P. (2010). Review: LIF and IL11 in trophoblast-endometrial interactions during the establishment of pregnancy. Placenta 31(Suppl.), 99–104 10.1016/j.placenta.2009.12.027 20129664

[B19] Enver T. Soneji S. Joshi C. Brown J. Iborra F. Orntoft T. (2005). Cellular differentiation hierarchies in normal and culture-adapted human embryonic stem cells, Hum. Mol. Genet. 14, 3129–3140 10.1093/hmg/ddi345 16159889

[B20] Fathi A. Pakzad M. Taei A. Brink T. C. Pirhaji L. Ruiz G. (2009). Comparative proteome and transcriptome analyses of embryonic stem cells during embryoid body-based differentiation. Proteomics 9, 4859–4870 10.1002/pmic.200900003 19862760

[B21] Flottmann M. Scharp T. Klipp E. (2012). A stochastic model of epigenetic dynamics in somatic cell reprogramming. Front. Physiol. 3:216 10.3389/fphys.2012.00216 22754535 PMC3384084

[B22] Gabut M. Samavarchi-Tehrani P. Wang X. Slobodeniuc V. O'Hanlon D. Sung H. K. (2011). An alternative splicing switch regulates embryonic stem cell pluripotency and reprogramming. Cell 147, 132–146 10.1016/j.cell.2011.08.023 21924763

[B23] Garg A. Mohanram K. Di Cara A. De Micheli G. Xenarios I. (2009). Modeling stochasticity and robustness in gene regulatory networks. Bioinformatics 25, i101–i109 10.1093/bioinformatics/btp214 19477975 PMC2687968

[B24] Greber B. Lehrach H. Adjaye J. (2007a). Fibroblast growth factor 2 modulates transforming growth factor beta signaling in mouse embryonic fibroblasts and human ESCs (hESCs) to support hESC self-renewal. Stem Cells 25, 455–464 10.1634/stemcells.2006-0476 17038665

[B25] Greber B. Lehrach H. Adjaye J. (2007b). Silencing of core transcription factors in human EC cells highlights the importance of autocrine FGF signaling for self-renewal. BMC Dev. Biol. 7:46 10.1186/1471-213X-7-46 17506876 PMC1885259

[B26] Greber B. Lehrach H. Adjaye J. (2008). Control of early fate decisions in human ES cells by distinct states of TGFbeta pathway activity. Stem Cells Dev. 17, 1065–1077 10.1089/scd.2008.0035 18393632

[B27] Greber B. Wu G. Bernemann C. Joo J. Y. Han D. W. Ko K. (2010). Conserved and divergent roles of FGF signaling in mouse epiblast stem cells and human embryonic stem cells. Cell Stem Cell 6, 215–226 10.1016/j.stem.2010.01.003 20207225

[B28] Hanna J. Cheng A. W. Saha K. Kim J. Lengner C. J. Soldner F. (2010). Human embryonic stem cells with biological and epigenetic characteristics similar to those of mouse ESCs. Proc. Natl. Acad. Sci. U.S.A. 107, 9222–9227 10.1073/pnas.1004584107 20442331 PMC2889088

[B29] Huang D. W. Sherman B. T. Lempicki R. A. (2009a). Bioinformatics enrichment tools: paths toward the comprehensive functional analysis of large gene lists. Nucleic Acids Res. 37, 1–13 10.1093/nar/gkn923 19033363 PMC2615629

[B30] Huang D. W. Sherman B. T. Lempicki R. A. (2009b). Systematic and integrative analysis of large gene lists using DAVID bioinformatics resources. Nat. Protoc. 4, 44–57 10.1038/nprot.2008.211 19131956

[B31] James D. Levine A. J. Besser D. Hemmati-Brivanlou A. (2005). TGFbeta/activin/nodal signaling is necessary for the maintenance of pluripotency in human embryonic stem cells. Development 132, 1273–1282 10.1242/dev.01706 15703277

[B32] Josephson R. Ording C. J. Liu Y. Shin S. Lakshmipathy U. Toumadje A. (2007). Qualification of embryonal carcinoma 2102Ep as a reference for human embryonic stem cell research. Stem Cells 25, 437–446 10.1634/stemcells.2006-0236 17284651

[B33] Jung M. Peterson H. Chavez L. Kahlem P. Lehrach H. Vilo J. (2010). A data integration approach to mapping OCT4 gene regulatory networks operative in embryonic stem cells and embryonal carcinoma cells. PLoS ONE 5:e10709 10.1371/journal.pone.0010709 20505756 PMC2873957

[B34] Kalmar T. Lim C. Hayward P. Munoz-Descalzo S. Nichols J. Garcia-Ojalvo J. (2009). Regulated fluctuations in nanog expression mediate cell fate decisions in embryonic stem cells. PLoS Biol. 7:e1000149 10.1371/journal.pbio.1000149 19582141 PMC2700273

[B35] Kanehisa M. Goto S. Sato Y. Furumichi M. Tanabe M. (2012). KEGG for integration and interpretation of large-scale molecular data sets. Nucleic Acids Res. 40, D109–D114 10.1093/nar/gkr988 22080510 PMC3245020

[B36] Kang H. B. Kim J. S. Kwon H. J. Nam K. H. Youn H. S. Sok D. E. (2005). Basic fibroblast growth factor activates ERK and induces c-fos in human embryonic stem cell line MizhES1. Stem Cells Dev. 14, 395–401 10.1089/scd.2005.14.395 16137228

[B37] Kim J. Chu J. Shen X. Wang J. Orkin S. H. (2008). An extended transcriptional network for pluripotency of embryonic stem cells. Cell 132, 1049–1061 10.1016/j.cell.2008.02.039 18358816 PMC3837340

[B38] Kunath T. Saba-El-Leil M. K. Almousailleakh M. Wray J. Meloche S. Smith A. (2007). FGF stimulation of the Erk1/2 signalling cascade triggers transition of pluripotent embryonic stem cells from self-renewal to lineage commitment. Development 134, 2895–2902 10.1242/dev.02880 17660198

[B39] Lanner F. Rossant J. (2010). The role of FGF/Erk signaling in pluripotent cells. Development 137, 3351–3360 10.1242/dev.050146 20876656

[B40] Lengner C. J. Gimelbrant A. A. Erwin J. A. Cheng A. W. Guenther M. G. Welstead G. G. (2010). Derivation of pre-X inactivation human embryonic stem cells under physiological oxygen concentrations. Cell 141, 872–883 10.1016/j.cell.2010.04.010 20471072

[B41] Levenstein M. E. Ludwig T. E. Xu R. H. Llanas R. A. VanDenHeuvel-Kramer K. Manning D. (2006). Basic fibroblast growth factor support of human embryonic stem cell self-renewal. Stem Cells 24, 568–574 10.1634/stemcells.2005-0247 16282444 PMC4615709

[B42] Li Y. Powell S. Brunette E. Lebkowski J. Mandalam R. (2005). Expansion of human embryonic stem cells in defined serum-free medium devoid of animal-derived products. Biotechnol. Bioeng. 91, 688–698 10.1002/bit.20536 15971228

[B43] Li W. Wei W. Zhu S. Zhu J. Shi Y. Lin T. (2009). Generation of rat and human induced pluripotent stem cells by combining genetic reprogramming and chemical inhibitors, Cell Stem Cell 4, 16–19 10.1016/j.stem.2008.11.014 19097958

[B44] Lister R. Pelizzola M. Dowen R. H. Hawkins R. D. Hon G. Tonti-Filippini J. (2009). Human DNA methylomes at base resolution show widespread epigenomic differences. Nature 462, 315–322 10.1038/nature08514 19829295 PMC2857523

[B45] Ludwig T. E. Levenstein M. E. Jones J. M. Berggren W. T. Mitchen E. R. Frane J. L. (2006). Derivation of human embryonic stem cells in defined conditions. Nat. Biotechnol. 24, 185–187 10.1038/nbt1177 16388305

[B46] Macarthur B. D. Ma'ayan A. Lemischka I. R. (2009). Systems biology of stem cell fate and cellular reprogramming. Nat. Rev. Mol. Cell Biol. 10, 672–681 10.1038/nrm2766 19738627 PMC2928569

[B47] Matin M. M. Walsh J. R. Gokhale P. J. Draper J. S. Bahrami A. R. Morton I. (2004). Specific knockdown of Oct4 and beta2-microglobulin expression by RNA interference in human embryonic stem cells and embryonic carcinoma cells. Stem Cells 22, 659–668 10.1634/stemcells.22-5-659 15342930

[B48] Milo R. Shen-Orr S. Itzkovitz S. Kashtan N. Chklovskii D. Alon U. (2002). Network motifs: simple building blocks of complex networks. Science 298, 824–827 10.1126/science.298.5594.824 12399590

[B49] Muller F. J. Laurent L. C. Kostka D. Ulitsky I. Williams R. Lu C. (2008). Regulatory networks define phenotypic classes of human stem cell lines. Nature 455, 401–405 10.1038/nature07213 18724358 PMC2637443

[B50] Nandurkar H. H. Hilton D. J. Nathan P. Willson T. Nicola N. Begley C. G. (1996). The human IL-11 receptor requires gp130 for signalling: demonstration by molecular cloning of the receptor. Oncogene 12, 585–593 8637716

[B51] Nandurkar H. H. Robb L. Begley C. G. (1998). The role of IL-II in hematopoiesis as revealed by a targeted mutation of its receptor. Stem Cells 16(Suppl. 2), 53–65 10.1002/stem.5530160708 11012177

[B52] Nichols J. Silva J. Roode M. Smith A. (2009). Suppression of Erk signalling promotes ground state pluripotency in the mouse embryo. Development 136, 3215–3222 10.1242/dev.038893 19710168 PMC2739140

[B53] R Development Core Team. (2011). R: A Language and Environment for Statistical Computing. Vienna: R Foundation for Statistical Computing ISBN 3-900051-07-0.

[B54] Remy E. Ruet P. Mendoza L. Thieffry D. Chaouiya C. (2006). From logical regulatory graphs to standard petri nets: dynamical roles and functionality of feedback citrcuits, in LNCS, (Berlin; Heidelberg: Springer-Verlag), 56–72 10.1007/11905455_3

[B55] Rosler E. S. Fisk G. J. Ares X. Irving J. Miura T. Rao M. S. (2004). Long-term culture of human embryonic stem cells in feeder-free conditions. Dev. Dyn. 229, 259–274 10.1002/dvdy.10430 14745951

[B56] Sato N. Meijer L. Skaltsounis L. Greengard P. Brivanlou A. H. (2004). Maintenance of pluripotency in human and mouse embryonic stem cells through activation of Wnt signaling by a pharmacological GSK-3-specific inhibitor. Nat. Med. 10, 55–63 10.1038/nm979 14702635

[B57] Singh A. M. Reynolds D. Cliff T. Ohtsuka S. Mattheyses A. L. Sun Y. (2012). Signaling network crosstalk in human pluripotent cells: a Smad2/3-regulated switch that controls the balance between self-renewal and differentiation. Cell Stem Cell 10, 312–326 10.1016/j.stem.2012.01.014 22385658 PMC3294294

[B58] Sudheer S. Bhushan R. Fauler B. Lehrach H. Adjaye J. (2012). FGF inhibition directs BMP4-mediated differentiation of human embryonic stem cells to syncytiotrophoblast. Stem Cells Dev. 21, 2987–3000 10.1089/scd.2012.0099 22724507 PMC3475151

[B59] Takahashi K. Yamanaka S. (2006). Induction of pluripotent stem cells from mouse embryonic and adult fibroblast cultures by defined factors. Cell 126, 663–676 10.1016/j.cell.2006.07.024 16904174

[B60] Tesar P. J. Chenoweth J. G. Brook F. A. Davies T. J. Evans E. P. Mack D. L. (2007). New cell lines from mouse epiblast share defining features with human embryonic stem cells. Nature 448, 196–199 10.1038/nature05972 17597760

[B61] Thomson J. A. Itskovitz-Eldor J. Shapiro S. S. Waknitz M. A. Swiergiel J. J. (1998). Embryonic stem cell lines derived from human blastocysts. Science 282, 1145–1147 10.1126/science.282.5391.1145 9804556

[B62] Timmermann A. Pflanz S. Grotzinger J. Kuster A. Kurth I. Pitard V. (2000). Different epitopes are required for gp130 activation by interleukin-6, oncostatin M and leukemia inhibitory factor. FEBS Lett. 468, 120–124 10.1016/S0014-5793(00)01205-9 10692570

[B63] Vallier L. Alexander M. Pedersen R. A. (2005). Activin/Nodal and FGF pathways cooperate to maintain pluripotency of human embryonic stem cells. J. Cell. Sci. 118, 4495–4509 10.1242/jcs.02553 16179608

[B64] Vallier L. Touboul T. Brown S. Cho C. Bilican B. Alexander M. (2009). Signaling pathways controlling pluripotency and early cell fate decisions of human induced pluripotent stem cells. Stem Cells 27, 2655–2666 10.1002/stem.199 19688839

[B65] Venables W. N. Ripley B. D. (2002). Modern Applied Statistics with S, 4th Edn. New York, NY: Springer ISBN 0-387-95457-0. 10.1007/978-0-387-21706-2

[B66] Wang J. Rao S. Chu J. Shen X. Levasseur D. N. Theunissen T. W. (2006). A protein interaction network for pluripotency of embryonic stem cells. Nature 444, 364–368 10.1038/nature05284 17093407

[B67] Wang L. Li L. Menendez P. Cerdan C. Bhatia M. (2005). Human embryonic stem cells maintained in the absence of mouse embryonic fibroblasts or conditioned media are capable of hematopoietic development. Blood 105, 4598–4603 10.1182/blood-2004-10-4065 15718421

[B68] Wang L. Schulz T. C. Sherrer E. S. Dauphin D. S. Shin S. Nelson A. M. (2007). Self-renewal of human embryonic stem cells requires insulin-like growth factor-1 receptor and ERBB2 receptor signaling. Blood 110, 4111–4119 10.1182/blood-2007-03-082586 17761519 PMC2190616

[B69] Wang Y. Xu Z. Jiang J. Xu C. Kang J. Xiao L. (2013). Endogenous miRNA sponge lincRNA-RoR regulates Oct4, Nanog, and Sox2 in human embryonic stem cell self-renewal. Dev. Cell 25, 69–80 10.1016/j.devcel.2013.03.002 23541921

[B70] Wernig M. Meissner A. Foreman R. Brambrink T. Ku M. Hochedlinger K. (2007). *In vitro* reprogramming of fibroblasts into a pluripotent ES-cell-like state. Nature 448, 318–324 10.1038/nature05944 17554336

[B71] Won K. J. Xu Z. Zhang X. Whitaker J. W. Shoemaker R. Ren B. (2012). Global identification of transcriptional regulators of pluripotency and differentiation in embryonic stem cells. Nucleic Acids Res. 40, 8199–8209 10.1093/nar/gks584 22730289 PMC3458541

[B72] Xu C. Inokuma M. S. Denham J. Golds K. Kundu P. Gold J. D. (2001). Feeder-free growth of undifferentiated human embryonic stem cells. Nat. Biotechnol. 19, 971–974 10.1038/nbt1001-971 11581665

[B73] Xu H. Baroukh C. Dannenfelser R. Chen E. Y. Tan C. M. Kou Y. (2013). ESCAPE: database for integrating high-content published data collected from human and mouse embryonic stem cells. Database (Oxford) 2013:bat045 10.1093/database/bat045 23794736 PMC3689438

[B74] Xu R. H. Chen X. Li D. S. Li R. Addicks G. C. Glennon C. (2002). BMP4 initiates human embryonic stem cell differentiation to trophoblast. Nat. Biotechnol. 20, 1261–1264 12426580 10.1038/nbt761

[B75] Xu C. Rosler E. Jiang J. Lebkowski J. S. Gold J. D. O'Sullivan C. (2005a). Basic fibroblast growth factor supports undifferentiated human embryonic stem cell growth without conditioned medium. Stem Cells 23, 315–323 10.1038/nbt761 15749926

[B76] Xu R. H. Peck R. M. Li D. S. Feng X. Ludwig T. Thomson J. A. (2005b). Basic FGF and suppression of BMP signaling sustain undifferentiated proliferation of human ES cells. Nat. Methods 2, 185–190 10.1038/nmeth744 15782187

[B77] Xu R. H. Sampsell-Barron T. L. Gu F. Root S. Peck R. M. Pan G. (2008). NANOG is a direct target of TGFbeta/activin-mediated SMAD signaling in human ESCs. Cell Stem Cell 3, 196–206 10.1016/j.stem.2008.07.001 18682241 PMC2758041

[B78] Ying Q. L. Wray J. Nichols J. Batlle-Morera L. Doble B. Woodgett J. (2008). The ground state of embryonic stem cell self-renewal. Nature 453, 519–523 10.1038/nature06968 18497825 PMC5328678

[B79] Yu J. Vodyanik M. A. Smuga-Otto K. Antosiewicz-Bourget J. Frane J. L. Tian S. (2007). Induced pluripotent stem cell lines derived from human somatic cells. Science 318, 1917–1920 10.1126/science.1151526 18029452

