# Supplementary material for: Qualitative modeling identifies IL-11 as a novel regulator in maintaining self-renewal in human pluripotent stem cells
Source: Front Physiol. 2013 Oct 28;4:303. doi: 10.3389/fphys.2013.00303 (PMC3809568; doi:10.3389/fphys.2013.00303)
Supplement: Supplementary Figure 1 — Raw data of initial alkaline phosphatase screen of predicted candidate genes for 3 days. Shown are four 12 well plates, each testing various concentrations of the predicted candidate cytokines or inhibitors of the respective pathway. Top left, WNT3A 1–100 ng/ml. Top right, Chalcone4 (inhibiting the CXCL12—CXCR7 axis) 10 nM–10 μM. Bottom left, IL-11 1–500 ng/ml. Bottom right, SP600125 (inhibiting JNK which in turn fails to activate c-JUN) tested at concentrations ranging from 0.1 to 50 μM. Pink arrows show DMSO control, red arrows show water control and the green arrows the SP600125 at 50 μM. [file Presentation1.PDF]

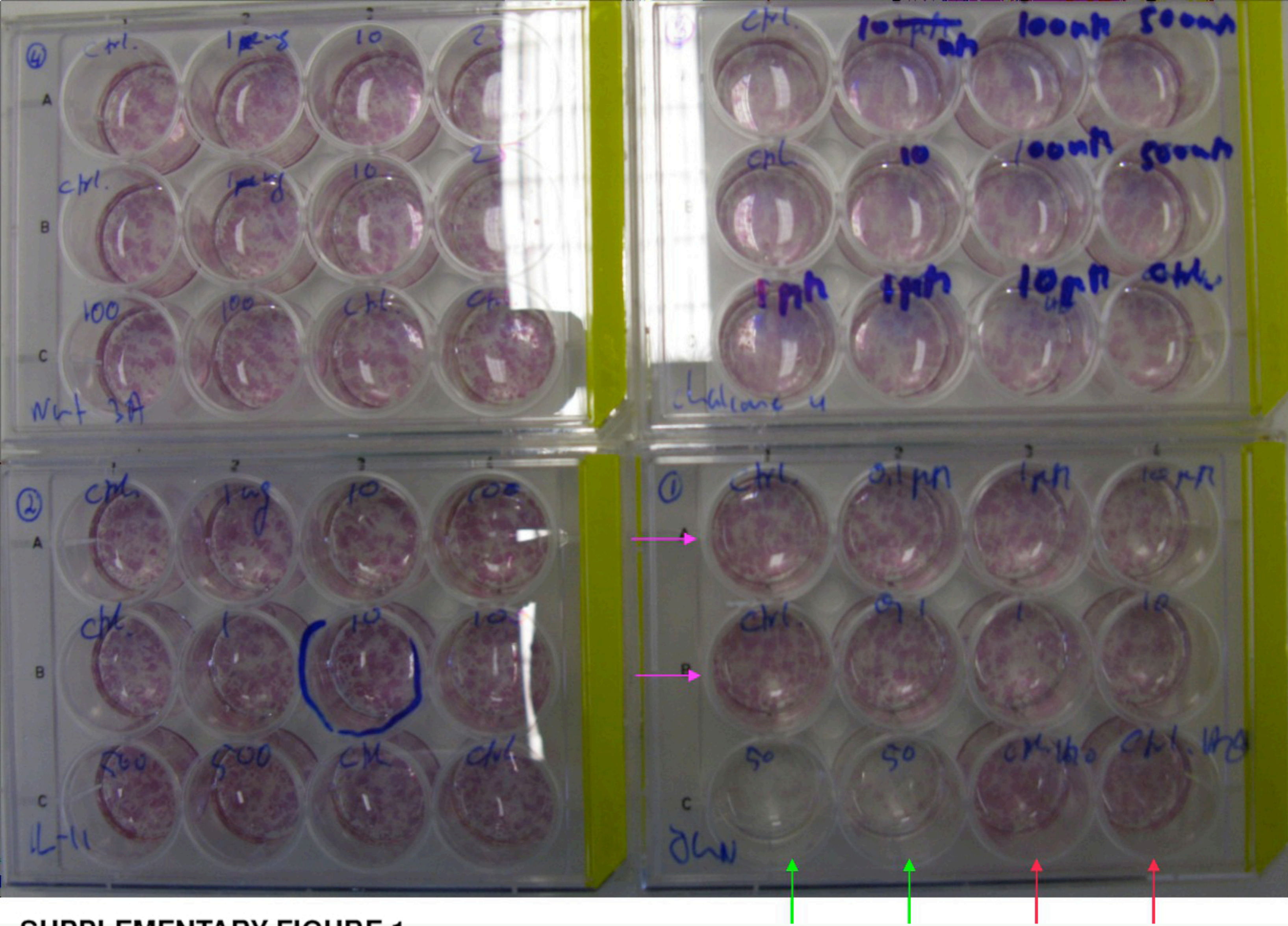

SUPPLEMENTARY FIGURE 1

Raw data of initial alkaline phosphatase screen of predicted candidate genes for 3 days. Shown are four 12 well plates, each testing various concentrations of the predicted candidate cytokines or inhibitors of the respective pathway. Top left, WNT3A 1ng/ml - 100ng/ml. Top right, Chalcone4 (inhibiting the CXCL12 - CXCR7 axis) 10nM - 10μM. Bottom left, IL-11 1ng/ml - 500ng/ml. Bottom right, SP600125 (inhibiting JNK which in turn fails to activate c-JUN) tested at concentrations ranging from 0.1μM - 50μM. Pink arrows show DMSO control, red arrows show water control and the green arrows the SP600125 at 50μM.
